# Tools of the trade: plasmid repositories and standardized plasmid manipulation for molecular and synthetic biology

**DOI:** 10.1111/1751-7915.14012

**Published:** 2022-02-08

**Authors:** Stephen K. Dolan, Miguel A. Matilla

**Affiliations:** ^1^ School of Biological Sciences Georgia Institute of Technology Atlanta GA 30332 USA; ^2^ Center for Microbial Dynamics and Infection Georgia Institute of Technology Atlanta GA 30332 USA; ^3^ Department of Biotechnology and Environmental Protection Estación Experimental del Zaidín Consejo Superior de Investigaciones Científicas Prof. Albareda 1 Granada 18008 Spain

## Abstract

Plasmids are extrachromosomal genetic elements capable of autonomous replication within a host cell. They play a key role in bacterial ecology and evolution, facilitating the mobilization of accessory genes by horizontal gene transfer. Crucially, plasmids also serve as valuable tools in modern molecular biology. Here, we highlight recent articles aimed at implementing standardized plasmid assembly techniques and plasmid repositories to promote open science as well as to improve experimental reproducibility across laboratories. Research focused on assisting these fundamental aims is a further step towards improving standardization in molecular and synthetic biology.

## Graphical abstract

The availability of plasmid repositories, together with improved DNA assembly techniques for the generation of recombinant plasmids, is accelerating the development and reproducibility of molecular biology, synthetic biology and biotechnology approaches.

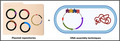

Plasmids are extrachromosomal independently replicating genetic elements and regular features in bacterial genomes. Indeed, the number of naturally occurring bacterial plasmids continues to grow rapidly with the advancement of new sequencing technologies. Currently, there are more than 34 000 bacterial plasmids listed in the NCBI Reference Sequence (RefSeq) database; which represents an almost twofold increase within the 2020–2021 period (Schmartz *et al*., 2021). Plasmids vary enormously in size, DNA topology (e.g. circular or linear) and copy number, among other features. In addition to encoding functions essential for maintenance, replication and conjugation, plasmids often carry genes that confer important traits for bacterial ecology and evolution, including antibiotic resistance, degradation of recalcitrant compounds and biosynthesis of toxins and secondary metabolites, among others (Norman *et al*., 2009; Rankin *et al*., 2011; Rodríguez‐Beltrán *et al*., 2021). However, despite their important roles in bacterial survival and evolution, plasmids impose a metabolic burden on the carrier bacteria that often results in a reduced bacterial fitness (Brockhurst and Harrison, 2021; Rodríguez‐Beltrán *et al*., 2021). There is a growing body of experimental data revealing that plasmid genes evolve differentially from chromosomal genes (Rodríguez‐Beltrán *et al*., 2021), which makes them key target systems for the study of bacterial evolutionary processes.

Plasmid vectors are the basis of recombinant DNA technology and their ongoing development continues to revolutionize molecular and synthetic biology (Nora *et al*., 2019; Damalas *et al*., 2020; Seco and Fernández, 2021; Vo *et al*., 2021). Notably, plasmid‐based expression systems are key for biotechnological processes aimed at the industrial production of, for example, organic compounds (e.g. vitamins, sugars, amino acids, etc.), enzymes, biofuels, biopolymers, drugs, antibodies and vaccines (Kroll *et al*., 2010; Prazeres and Monteiro, 2014; Huertas and Matilla, 2020) as well as for the biodegradation of contaminants (Bhatt *et al*., 2021). In this context, to maximize scientific progress and encourage open science and reproducibility, an increasing number of plasmid repositories are accessible to the scientific community. The volume of plasmids stored in these repositories has grown greatly in recent years and three of the largest repositories worldwide, iGEM, DNASU and Addgene, store over 300 000 plasmids and distribute them to researchers over the globe (Timmons and Densmore, 2020). However, the capacity of these repositories to provide plasmids with altered structure and functionality represents a bottleneck to the efficient use of these vector collections.

Addressing the issue of standardizing plasmid engineering was central to the creation of the SEVA (Standard European Vector Architecture) compositional standard, a set of rules for the physical assembly of three basic components of plasmid vectors (origin of replication, selection marker and a ‘cargo module’), which are interconnected by invariant sequences. These rules were bolstered through the creation of the SEVA platform (http://seva‐plasmids.com/), a web‐based resource and clone repository designed to assist users regarding optimal plasmid vector selection, and the SEVA database (SEVA‐DB), which allows users to customize plasmid design for their specific needs, deciding on the optimal configuration of the above basic components. This valuable resource has been used to answer a plethora of fundamental and applied questions in prokaryotic biology – primarily in Gram‐negative bacteria but also in Gram‐positive bacterial hosts (Silva‐Rocha *et al*., 2013; Damalas *et al*., 2020; Garcia‐Gutiérrez et al., 2020; Martínez‐García *et al*., 2020).

SEVA vectors have been widely used in *Escherichia coli* and in the *Pseudomonas putida* biotechnology research community (Silva‐Rocha *et al*., 2013; Damalas *et al*., 2020; Garcia‐Gutiérrez *et al*., 2020), but their potential in research involving alternative hosts has not been fully realized. In a recent *Microbial Biotechnology* report, Lammens and co‐workers use the established SEVA‐vector backbone to develop ‘SEVAtile’, a highly efficient, Type IIs restriction enzyme‐based assembly method which enables the rapid and reproducible assembly of genetic parts (tiles) to create genetic constructs optimized for different biotechnological and synthetic biology applications (Lammens *et al*., 2022). This is achieved by adding part‐specific position tags of four nucleotides to the flanking regions of each building block, which forces the tiles to ligate in the desired order. Two key advantages of this approach are the easy randomization of parts in a genetic construct and tile reuse. Shuffling libraries of building blocks, when in combination with a suitable high‐throughput screening method, could allow one to explore a vast sequence space of variants. This approach stems from the ‘VersaTile’ DNA assembly technique, which was recently used to rapidly construct a combinatorial library of approximately 10 000 lysin variants in *E. coli* for antibacterial screening (Gerstmans *et al*., 2020).

As a proof of concept, Lammens *et al*. (2022) used the ‘SEVAtile’ approach to generate 14 different destination vectors containing up to six building blocks. This assembly method was proved to be highly efficient (i.e. average transformation yields over 5 × 10^4^ CFU per microgram of transformed DNA) and the functionality of the final vectors was demonstrated in *P. putida* KT2440 and *P. aeruginosa* PAO1 (Lammens *et al*., 2022). Remarkably, the authors used this approach to generate a three‐vector system consisting of ‘pSTDesX’ (a XylS/*Pm* expression system), ‘pSTDesR’ (a RhaRS/*PrhaB* expression system) and ‘pBGDes’ (a Tn7 chromosomally integrated, constitutively expressed system) to independently co‐express three different proteins in KT2440 and PAO1. Although expression was achieved in both hosts, distinct expression responses were noted for these two pseudomonads (Lammens *et al*., 2022), which may be associated to the different physiology and metabolic architectures of PAO1 and KT2440 (Kohlstedt and Wittmann, 2019). This work highlights the potential of SEVA vectors and innovative DNA assembly techniques to the synthetic and molecular biology community.

Maintaining large collections of standardized plasmids can result in a few surprising challenges. In a recent report published in *Microbial Biotechnology*, Brkljacic *et al*. (2021) investigated the integration of bacterial mobile genetic elements (MGE) in plasmid stocks distributed by two different repositories: the ‘Arabidopsis Biological Resource Centre’ (ABRC; https://abrc.osu.edu/) and ‘Addgene’ (http://www.addgene.org/). These two repositories rely on donations from the scientific community and store more than 460 000 and 106 000 plasmids, respectively. The work by Brkljacic *et al*. was motivated by the observation that several deposited plasmids had unexpected restriction patterns. All deposited plasmids in ABRC and Addgene passed exhaustive quality controls that included: (i) the analysis by restriction endonuclease digestion of various individual colonies (ABRC); or (ii) plasmid sequencing and the annotation of transposable elements like insertion sequences (IS) (Addgene). However, the re‐analysis of various plasmid stocks by restriction digestion, PCR and sequencing revealed the insertion of undesired IS elements in plasmids with diverse backbones. This IS acquisition was found to occur at both prior to stock deposition and during host culturing at repositories (Brkljacic *et al*., 2021). Growth‐based experimentation allowed the authors to propose that IS‐insertion conferred a selective advantage over plasmids with no IS elements as well as to demonstrate that the use of MGE‐free *Escherichia coli* hosts for plasmid propagation prevents the insertion of IS elements (Brkljacic *et al*., 2021). The type and frequency of IS elements transposed during plasmid generation or storage was determined after the analysis of nearly 48 000 sequences of plasmids deposited at Addgene. The results showed that ~ 1.1% of these plasmids contained at least one IS insertion and six different types of IS elements were identified, with IS*1A* and IS*10R* being by far the most frequent (Brkljacic *et al*., 2021). Although the presence of unexpected MGEs has not resulted in any reported negative effect on plasmid function, these IS insertions could in theory alter plasmid functionality and stability, thus, hampering standardization efforts.

These two studies highlight the enormous potential and some challenges that lie ahead for plasmid research and standardization. ‘SEVAtile’ upholds the clear advantages of *á la carte* plasmid design and accessibility, while also facilitating extensive variation within a plasmid assembly. It will be exciting to see how this approach will be used to probe *Pseudomonas* core architecture, and to expand the capability of different hosts in synthetic biology applications. In contrast, the use of IS‐free hosts and additional controls will hopefully slow unwanted variation in plasmid sequences during plasmid construction and propagation. Future research in this field will facilitate the generation of new molecular tools for the use in a broad spectrum of model bacteria, including non‐standard hosts.

## Conflict of interest

The authors declare that there is no conflict of interest.
